# Regarding: “Are medical students interested in research? – Students’ attitudes towards research”

**DOI:** 10.1080/07853890.2022.2108131

**Published:** 2022-08-05

**Authors:** Eirinaios Tsiartas, Dimitra Kontopyrgou

**Affiliations:** School of Medicine, Faculty of Health Sciences, Aristotle University of Thessaloniki, Thessaloniki, Greece

Dear Editor,

We read with great interest the cross-sectional study by Sobczuk et al. [1] regarding medical students and their perceptions of research. The authors provided an analysis with encouraging prospects for the future. Nevertheless, we would like to highlight certain matters that we believe should be taken into consideration and discussed furtherly.

The declining numbers of physician-investigators and the alarming relevant consequences are an acknowledged issue, first raised by Wyngaarden [2] more than forty years ago, but it is not yet resolved. The essentiality of medical breakthroughs is unequivocal, and it is very promising that medical students express a high interest in research during their medical studies [1,3]. Naturally, motives vary vastly; from simple curiosity to aspirations for an academic career or a competitive residency program [3]. Therefore, the correlation of that interest with the possibility of a dedicated career in research should be done with caution. In addition, previous studies indicated that eventual career choice may differ from initial choices, or even be done years after graduation [4]. Subsequently, comparisons between the limited sample group of second and fifth-year students of a six-year medical program, might have produced ambiguous conclusions that do not fully reflect the effect the advancement of their studies had on their perceptions.

Moreover, students, especially younger ones, or in countries where medicine is not exclusively graduate entry (e.g. in the USA), often lack the necessary experience to thrive on their own. Hence, constructive supervision by an experienced mentor is an essential part of the equation. Its absence, especially when accompanied by financial constraints and demanding syllabi, results in a small proportion that actually attempts and succeeds in becoming a student-researcher [3]. Consequently, the approach should include a focus on the rationale behind the fact that some students express an early interest in research while others do not. Their personal background, motives, and perceived impediments should be a subject of inquiry. Specifically, students’ background and prior experience is a recognized factor that contributes to their perspective towards research [3,5]. The latter is not thoroughly addressed in the study by Sobczuk et al. [1], although it poses a potential target for future interventions.

Nonetheless, a tangible effect of the early commitment to research is an increased likelihood and higher success rate in pursuing an academic or research career later [3]. Thus, the implementation of coherent reforms will convert students’ interest into initiatives, which will provide better prospects for evolving them into medical researchers.

In conclusion, medical students’ eagerness to engage in research is evident, and efforts should be made to cultivate their potentials as researchers as soon as possible. Cross-sectional studies, such as the one by Sobczuk et al. [1], should generate the design of relevant prospective studies to assess interventions and define an effective framework. Ultimately, we emphasize the necessity for a broader approach that will expand the tendency of early involvement in research.

## Data Availability

Not applicable. No new data were created or analysed for this article.
